# A challenging case of an intraorbital foreign body in a child: A case report

**DOI:** 10.1016/j.amsu.2022.103471

**Published:** 2022-03-04

**Authors:** Nadia Ben Abdessalem, Nesrine Zaafrane, Ahmed Jabri, Fedi Sahraoui, Atf Ben Abderrazek, Anis Mahjoub, Arij Dlensi, Anis Ayadi, Hachemi Mahjoub, Ahmed Mahjoub

**Affiliations:** a Ophthalmology Department, Farhat Hached University Hospital of Sousse, Tunisia; b Maxillofacial Surgery Department, Sahloul University Hospital of Sousse, Tunisia; c Faculty of Medicine of Sousse, University of Sousse, Tunisia

**Keywords:** Case report, Child, Intraorbital foreign body, Anterior orbitotomy, CT scan

## Abstract

Apart from congenital causes, orbital trauma is a leading cause of unilateral vision loss in children.

We report the case of a 2-year-old child who was victim of an orbital trauma of the right eye caused by a ballpoint pen. He consulted us the day after the trauma with significant palpebral edema making the examination difficult. An emergency CT scan of the orbit and brain showed the presence of a right intraorbital foreign body.

The patient underwent removal of the foreign body by an anterior orbitotomy with general antibiotic therapy and a simple postoperative course.

Penetrating trauma to the orbit should raise the suspicion of the presence of a foreign body. A CT scan should be performed to specify its location. The extraction of the foreign body can be a challenge that requires an experienced surgical team.

## Highlights

• Penetrating trauma to the orbit should raise the suspicion of the presence of a foreign body.
• CT reconstruction is necessary to specify the exact location of the foreign body
• The extraction of the foreign body can be a challenge that requires an experienced surgical team.

## 1 Introduction and importance

Apart from congenital causes, orbital trauma is a leading cause of unilateral vision loss in children [1].

Intraorbital foreign bodies usually occur after high-speed orbital trauma [2].

If left in place, the intra-orbital foreign body would be a source of serious complications, particularly infectious ones.

We report the case of a 2-year-old patient victim of orbital trauma with a CT scan showing an intraorbital foreign body.

This case report has been reported in line with the SCARE criteria [3].

## 2 Case presentation

Our patient was a 2-year-old child with no past medical history, who presented to the emergency department with a right orbital trauma.

The interrogation disclosed the notion of a direct trauma caused by a fall of the child from his own height on a perforating object: a ballpoint pen.

In general, the patient was conscious and well oriented.

**Fig. 1:**
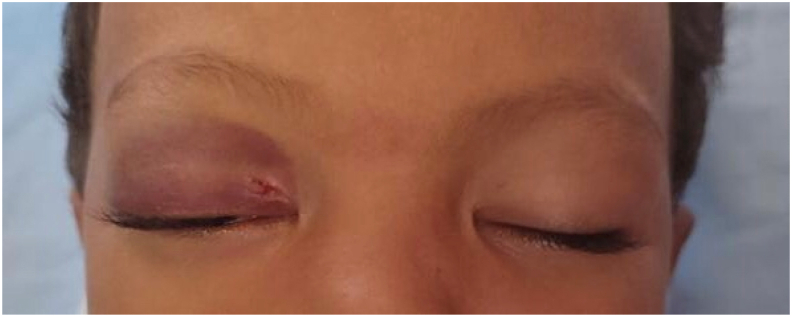
Frontal photograph of the child showing edema, ecchymosis, and a right upper palpebral wound.

The initial ophthalmologic examination revealed significant palpebral edema of the right eye, which restricted palpebral opening. In addition, we noted the presence of a 5 mm wound on the inner third of the upper eyelid (Fig. 1).

A surgical exploration of the right eye, carried out in emergency, did not find any visible foreign body and no wound of the eyeball. Moreover, the examination under general anesthesia showed a calm anterior segment and a fundus without abnormalities.

**Fig. 2:**
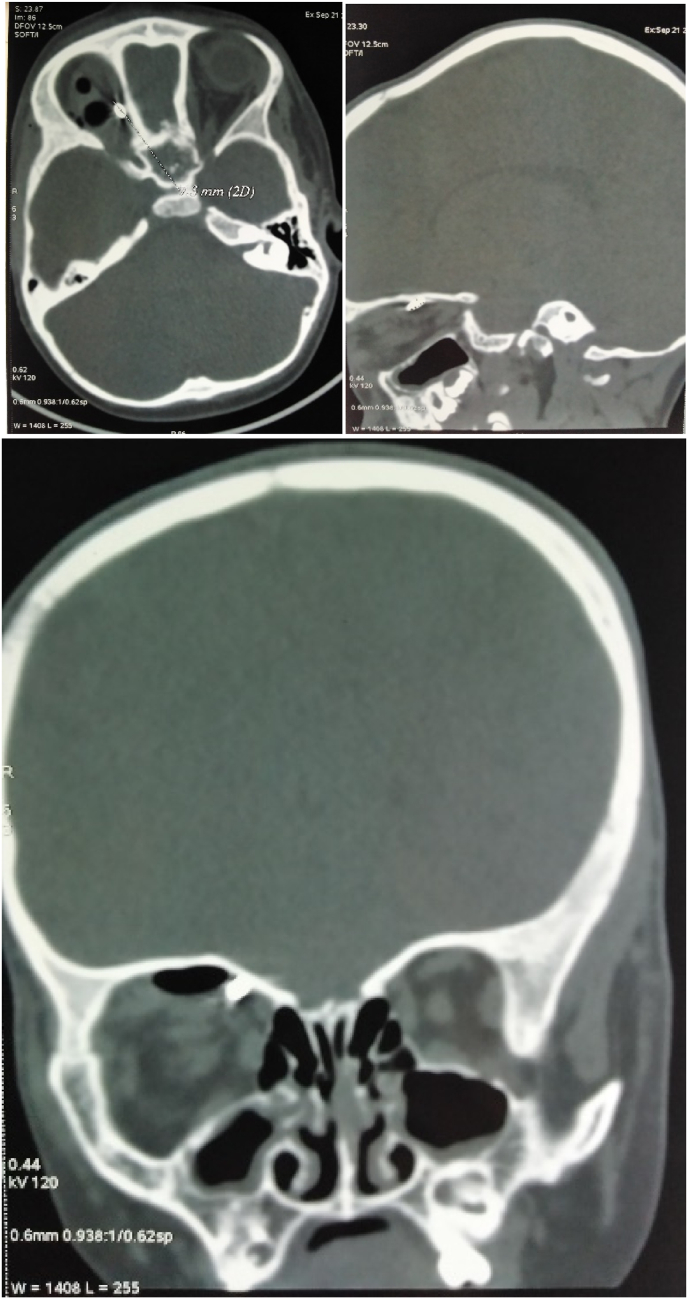
Non-injected CT image in coronal, sagittal and frontal sections showing a metallic foreign body contacting the upper wall of the orbit with pneumobitis.

An orbito-cerebral computed tomography (CT) scan without injection of contrast product was performed and proved the existence of a 7-mm right intraorbital metallic foreign body within the upper wall of the orbit, lateralized internally, opposite the homolateral superior oblique muscle, associated with a minimal pneumorbitis (Fig. 2).

During the child's hospitalization in our ophthalmology department, local and systemic antibiotic and corticosteroid therapy was started and the tetanus vaccination was up to date.

**Fig. 3:**
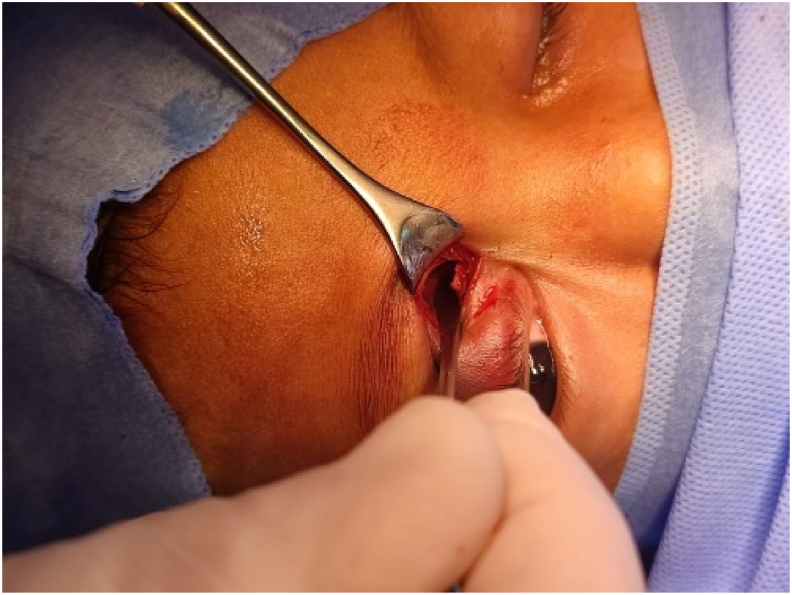
Operative view during anterior orbitotomy through the initial wound.

**Fig. 4:**
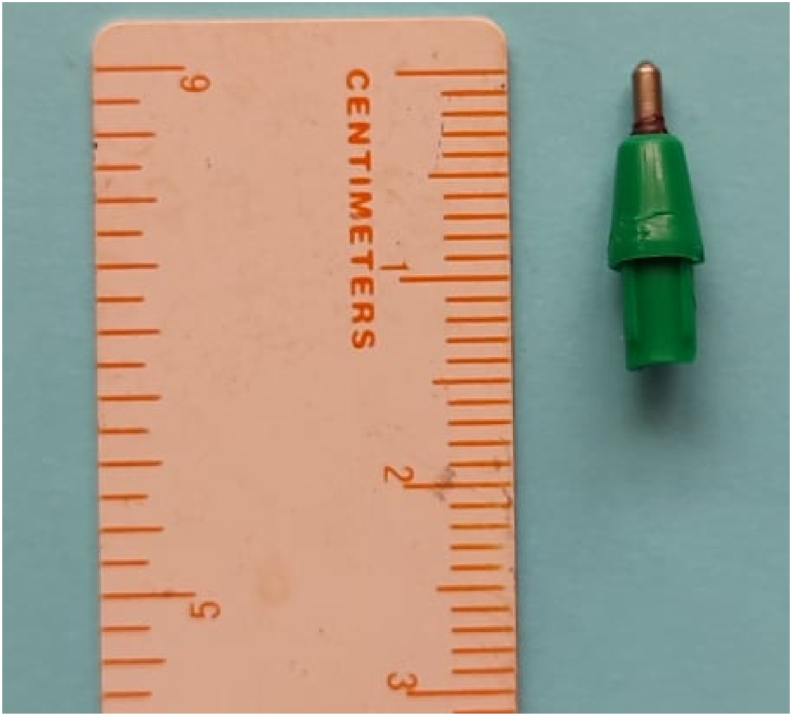
The metallo-plastic foreign body removed during surgery.

After parental permission, we performed, in collaboration with maxillofacial surgeons, an anterior orbitotomy under general anesthesia using the same foreign body penetration route (Fig. 3). The dissection allowed the extraction of a metallo-plastic foreign body, which was the tip of the pen (Fig. 4). The orbitotomy was then closed without any drainage and the postoperative course was simple.

**Fig. 5:**
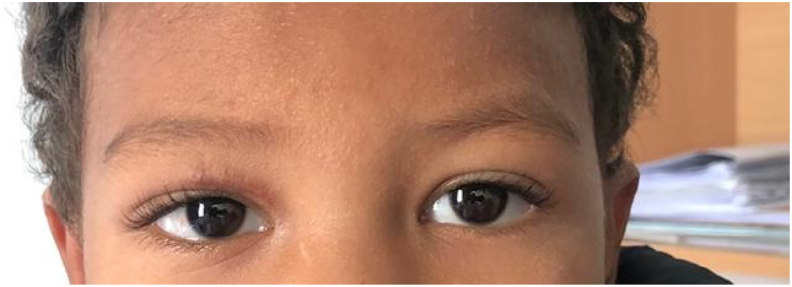
Frontal photograph of the child during the control examination.

The patient was discharged after six days of hospitalization and a follow-up examination, performed after one week of discharge, was without abnormalities with a closed and clean wound (Fig. 5).

This case report has been reported in line with the SCARE criteria [3].

## 3 Clinical discussion

Ocular trauma is an important cause of ocular morbidity in children and the incidence of intraorbital foreign bodies during orbital trauma is 2.9% [4].

Accidental falls are among the most frequent causes of pediatric ocular trauma, estimated at 37% [5].

An unrecognized foreign body can reveal itself with complications, even at a distance from the accident [6].

CT is the imaging method of choice for the detection of intraorbital foreign bodies. Magnetic resonance imaging (MRI) is not always available in emergency and is contraindicated once an intraorbital foreign body of metallic nature is suspected [7].

The best approach is to obtain thin axial CT slices in the range of 0.625–1.25mm, depending on the capabilities of the scanner [8].

In our case, we suspected the presence of an intraorbital foreign body in front of a significant palpebral edema and an ecchymosis with a cutaneous entrance.

It is recommended that all patients with intraorbital foreign body, organic or non-organic, receive appropriate tetanus prophylaxis and broad-spectrum antibiotics in collaboration with infectious disease specialists, even in the absence of apparent infection [9].

Surgical removal is guided by the nature and location of the foreign body in the orbit. It is generally recommended for all organic foreign bodies because of the higher risk of inflammation and infection. It is also indicated for intraorbital foreign bodies that impede the mobility of the globe or those complicated by infection and for exophthalmos or optic nerve compression [10].

Foreign bodies located posterior to the orbit have an increased risk of oculomotor dysfunction and optic neuropathy after surgical removal, whereas anteriorly placed foreign bodies are easier to remove, and removal of an anteriorly located metallic foreign body facilitates MRI [8].

Two types of inorganic foreign body require special attention: those made of copper with the risk of chalcosis and significant orbital inflammation and those made of iron with the risk of siderosis [9].

Extraction is also legitimate in the presence of a large and sharp foreign body, which was the surgical indication in our observation, especially since there was doubt about the exact nature of the foreign body [10].

The surgical approach chosen is the one that provides the best access. It can be performed either through the path of penetration of the foreign body or remotely [11] In our observation, we accessed the foreign body through the initial wound.

## 4 Conclusion

Penetrating trauma to the orbit should raise the suspicion of the presence of a foreign body. It is necessary to specify its exact location, thanks to CT reconstructions, because if it is not known, it can lead to serious complications, especially infectious ones. The extraction of the foreign body is to be discussed according to the clinical situation and can be a challenge that requires an experienced surgical team.

## Ethical approval

We further confirm that any aspect of the work covered in this manuscript that has involved human patients has been conducted with the ethical approval of all relevant bodies and that such approvals are acknowledged within the manuscript. IRB approval was obtained (required for studies and series of 3 or more cases) Written consent to publish potentially identifying information, such as details or the case and photographs, was obtained from the patient(s) or their legal guardian(s).

## Sources of funding

No funding was received for this work.

## Author contribution

Nadia Ben Abdesslem: writing the paper. Nesrine Zaafrane: data analysis. Ahmed Jabri: writing the paper. Fedi Sahraoui: collecting data. Atf Ben Abderrazek: study concept. Anis Mahjoub: study design. Aryj Dlensi: study design. Anis Ayadi: collecting data. Hachemi Mahjoub: correcting the final paper. Ahmed Mahjoub: correcting the final paper.

## Registration of Research Studies

1. Name of the registry:
2. Unique Identifying number or registration ID:
3. Hyperlink to your specific registration (must be publicly accessible and will be checked):

## Guarantor

Atf Ben Abderrazek atf.benabderrazek@gmail.com.

## Consent

“Written informed consent was obtained from the patient's legal guardian: The father, for publication of this case report and accompanying images. A copy of the written consent is available for review by the Editor-in-Chief of this journal on request”.

## Declaration of competing interest

No conflict of interest exists. We wish to confirm that there are no known conflicts of interest associated with this publication and there has been no significant financial support for this work that could have influenced its outcome.
